# Evaluation of pyocin activity and pyocin-related gene distribution in multidrug- and extensively drug-resistant *Pseudomonas aeruginosa* isolates from Thailand

**DOI:** 10.7717/peerj.21850

**Published:** 2026-10-21

**Authors:** Phatcharin Laosena, Punthira Suppaiboolkij, Pornpavee Nualnisachol, Daniel Walker, Jiraphan Premsuriya

**Affiliations:** 1 Princess Srisavangavadhana Faculty of Medicine, Chulabhorn Royal Academy, Bangkok, Thailand; 2 Faculty of Science, Kasetsart University, Bangkok, Thailand; 3 Strathclyde Institute of Pharmacy and Biomedical Sciences, University of Strathclyde, Glasgow, United Kingdom; 4 Research Center on Clinical and System Microbiology (RCSyM), Chulabhorn Royal Academy, Bangkok, Thailand

**Keywords:** Pyocins, Bacteriocins, *Pseudomonas aeruginosa*, Protein antibiotics, Antimicrobial resistance, Biofilm

## Abstract

*Pseudomonas aeruginosa* remains a major cause of healthcare-associated infections worldwide, with clinical management increasingly compromised by the emergence and dissemination of multidrug-resistant (MDR) and extensively drug-resistant (XDR) lineages. In this study, we evaluated the antibacterial and antibiofilm activity of purified S-type pyocins (AP41, SX1, SX2, G, S5, and M1) against a panel of 29 MDR or XDR clinical *P. aeruginosa* isolates collected from across Thailand in 2021–2022, integrating phenotypic data with comparative genomic analyses. Susceptibility screening using overlay spot assays identified AP41, SX1, and SX2 as the pyocins with the broadest activity. These pyocins exhibited potent killing activity across MDR and XDR isolates, including strains resistant to last-resort antibiotics such as carbapenems and colistin. Minimum inhibitory concentration assays demonstrated high antibacterial potency, with AP41 and SX1 achieving inhibitory activity at low concentrations within the microgram range. In biofilm eradication experiments, AP41, SX1, and SX2 produced dose-dependent reductions in the metabolic activity of established biofilms, indicating efficacy against both planktonic and biofilm-associated cells. Whole-genome analysis of a combined dataset comprising Thai clinical *P. aeruginosa* genomes retrieved from the National Center for Biotechnology Information (NCBI) Pathogen Detection database, together with genomes from isolates included in this study (*n* = 105), revealed that pyocin transporter genes were widely conserved, whereas repertoires of genes encoding pyocins and determinants of cognate immunity to them displayed marked heterogeneity and strong lineage association. Notably, the transporter gene associated with SX1 and SX2 uptake (*crtA*) was conserved across the genome dataset, while no genes conferring cognate immunity against SX1 or SX2 were identified. This suggested that SX1 and SX2 retain activity across a broad range of isolates. Collectively, these findings demonstrate that pyocins exhibit potent antibacterial and antibiofilm activity against MDR and XDR *P. aeruginosa*, underscoring their potential as candidates for the development of alternative antimicrobial therapies.

## Introduction

*Pseudomonas aeruginosa* is an important opportunistic pathogen in healthcare settings, with multidrug-resistant (MDR) strains posing therapeutic challenges (Hirsch & Tam, 2010). Globally, *P. aeruginosa* was among the five bacterial pathogens associated with the greatest number of deaths in 2019 (GBD 2019 Antimicrobial Resistance Collaborators, 2022). It is responsible for a broad spectrum of infections, including hospital-acquired and ventilator-associated pneumonia, bloodstream infections, urinary tract infections, burn and wound infections, and chronic respiratory infections in patients with cystic fibrosis or other immunocompromising conditions (Sathe et al., 2023). Therapeutic management is further complicated by the organism’s intrinsic resistance to multiple antimicrobial classes, including *β*-lactams, aminoglycosides, and fluoroquinolones, as well as its remarkable capacity to acquire additional resistance determinants through chromosomal mutation and horizontal gene transfer (Pachori, Gothalwal & Gandhi, 2019).

A key biological trait underpinning the persistence and clinical recalcitrance of *P. aeruginosa* is its ability to form structured biofilms on both biotic and abiotic surfaces, such as medical devices and host tissues (Thi, Wibowo & Rehm, 2020). Within biofilms, bacterial populations are embedded in a self-produced extracellular matrix that restricts antibiotic penetration, alters the local microenvironment, and promotes metabolic heterogeneity (Wei & Ma, 2013). Consequently, biofilm-associated *P. aeruginosa* is markedly less susceptible to antimicrobial agents and host immune responses, facilitating chronic infection, treatment failure, and recurrent disease (Tuon et al., 2022). This biofilm-based lifestyle also provides a favorable ecological niche for the selection and maintenance of resistant subpopulations, further obstructing successful antimicrobial therapy (Wei & Ma, 2013; Tuon et al., 2022).

Reflecting its substantial global disease burden and the progressive erosion of effective treatment options, *P. aeruginosa* was designated a high-priority pathogen on the World Health Organization (WHO) bacterial priority pathogen list (World Health Organization, 2024). This underscores the urgent need to develop novel antimicrobial agents and alternative therapeutic strategies that extend beyond conventional antibiotics. In Thailand, the public health impact of MDR and extensively drug-resistant (XDR) *P. aeruginosa* has become increasingly evident. National surveillance data and molecular epidemiological studies have documented widespread dissemination of resistant strains across the country and in diverse healthcare settings, particularly within intensive care units and tertiary-care hospitals (Palavutitotai et al., 2018; Yungyuen et al., 2021; Wankaew et al., 2026). Whole-genome sequencing analyses in Thailand have revealed the circulation of internationally recognized high-risk clones harboring complex arrays of resistance determinants, including carbapenemases and efflux-associated mutations, further highlighting the limitations of existing antimicrobial regimens and the need for innovative treatment approaches (Wankaew et al., 2026).

In response to the escalating antimicrobial resistance crisis, bacteriocins have attracted renewed interest as potential alternatives or adjuncts to conventional antibiotics. Bacteriocins are proteinaceous toxins produced by many bacteria that provide a competitive advantage to the producing strain by selectively eliminating closely related competitors occupying similar ecological niches and competing for limited resources. This narrow-spectrum activity distinguishes bacteriocins from many small-molecule antibiotics and is enabled by their highly specific targeting mechanisms (Preciado et al., 2016). Among Gram-negative pathogens, *P. aeruginosa* is notable for producing a diverse repertoire of bacteriocins known as pyocins. Pyocins are classically categorized into R-type, F-type, and S-type groups according to their structural organization and mechanisms of action (Ghequire & De Mot, 2014).

S-type pyocins are soluble, multi-domain protein toxins that mediate highly specific bactericidal activity against susceptible *P. aeruginosa* strains. S-type pyocins share structural and functional similarities with colicins of *Escherichia coli*, particularly in their cell entry mechanisms and cytotoxic modes of action (Parret & De Mot, 2002; Michel-Briand & Baysse, 2002). Most characterized S-type pyocins exert cytotoxic effects through nuclease activities targeting DNA, rRNA, or tRNA, whereas others act *via* pore formation or inhibition of peptidoglycan synthesis (Ghequire & De Mot, 2014). To prevent self-intoxication, pyocins are co-expressed with cognate immunity proteins that neutralize their cytotoxic domains (Michel-Briand & Baysse, 2002; Ghequire & De Mot, 2014). S-type pyocins exploit receptor-mediated entry mechanisms involving outer membrane components. Initial binding frequently occurs *via* common polysaccharide antigen (CPA), facilitating the accumulation of pyocin on the bacterial surface. Subsequent translocation across the outer membrane is commonly mediated by TonB-dependent transporters (TBDTs), which normally function in siderophore-mediated metal acquisition. Pyocins utilize these transport systems through receptor-binding domains and N-terminal TonB-binding motifs, enabling energy-dependent import *via* the TonB1–ExbB–ExbD complex (Behrens et al., 2020).

Importantly, the receptor-mediated uptake pathways and intracellular killing mechanisms of S-type pyocins differ fundamentally from those targeted by conventional antibiotics. This mechanistic divergence likely contributes to the retention of pyocin activity against isolates harboring established resistance mechanisms, including enzymatic drug inactivation, target modification, and efflux pump overexpression (Ghequire & De Mot, 2014). In addition, *in vitro* and *in vivo* studies have demonstrated that pyocins can mediate rapid and selective killing, significantly reduce bacterial burden in biofilm models, and improve outcomes in animal infection systems (Smith et al., 2012; Ghequire & De Mot, 2014; McCaughey et al., 2016; Six et al., 2021), highlighting their therapeutic potential.

Unlike small-molecule antibiotics, pyocins are protein-based antimicrobials that are inherently amenable to rational engineering. Advances in protein design and synthetic biology have enabled the modification of pyocin domains to enhance receptor specificity, expand strain coverage, improve stability, and optimize pharmacological properties (Ghequire & De Mot, 2014). Furthermore, the increasing clinical acceptance of protein-based therapeutics, as illustrated by monoclonal antibodies and enzyme replacement therapies, underscores the growing feasibility of deploying engineered proteins as antimicrobial agents (Dimitrov, 2012). Within this evolving therapeutic landscape, pyocins represent a promising platform for next-generation antimicrobials targeting MDR and XDR *P. aeruginosa*. These findings support further investigation of pyocin-based strategies as alternative or adjunct approaches to conventional antibiotic therapy.

Against this background, this study was implemented to evaluate the efficacy of pyocins against MDR and XDR *P. aeruginosa* isolates from Thailand in both planktonic and biofilm states, and to investigate the distribution of pyocin-associated genes within the Thai clinical *P. aeruginosa* population. Pyocin-mediated killing depends on the presence of compatible outer membrane transporters that enable cellular uptake, as well as the absence of cognate immunity proteins in target cells (Ghequire & De Mot, 2014). Accordingly, characterizing the distribution of transporter and immunity genes may help to predict pyocin susceptibility and identify pyocins able to kill a broad range of strains. Collectively, the phenotypic and genomic findings of this study support the potential development of pyocins as novel therapeutic agents against MDR and XDR *P. aeruginosa*.

## Materials & Methods

### *P. aeruginosa* strains

A total of 29 MDR or XDR *P. aeruginosa* clinical isolates collected in Thailand between 2021 and 2022 were obtained from the existing culture collection of the National Institute of Health, Thailand (Thai NIH). MDR was defined as resistance to at least three classes of antipseudomonal agents, whereas XDR was defined as non-susceptibility to at least one agent in all but two or fewer antimicrobial categories, including antipseudomonal penicillins, cephalosporins, carbapenems, fluoroquinolones, aminoglycosides, and polymyxins (Magiorakos et al., 2012). Isolates were selected from multiple geographical regions of Thailand (North, Central, Northeast, and South) to ensure broad representation of circulating pathogens from across the country. The whole-genome sequencing and genomic characterization of these isolates have been described previously (Wankaew et al., 2026).

### Pyocins

S-type pyocins AP41, SX1, SX2, G, S5, and M1 were purified according to previously published protocols (McCaughey et al., 2016; Premsuriya et al., 2023). Briefly, pyocin-encoding genes were cloned into pET expression plasmids and expressed in *E. coli* BL21 (DE3). Recombinant proteins were purified by nickel affinity chromatography followed by size-exclusion chromatography. Final purified pyocins were buffer-exchanged into 20 mM Tris–HCl and 200 mM NaCl (pH 7.5) and stored at −80 °C until use.

### Antimicrobial susceptibility testing

Antimicrobial susceptibility testing was performed using the disk diffusion method in accordance with the Clinical and Laboratory Standards Institute (CLSI) guidelines. The antibiotics evaluated by disk diffusion included piperacillin–tazobactam, ceftazidime, cefepime, aztreonam, imipenem, meropenem, gentamicin, amikacin, and ciprofloxacin. Colistin susceptibility was determined by broth microdilution minimum inhibitory concentration (MIC) testing in accordance with CLSI recommendations. All results were interpreted according to the breakpoints specified in the CLSI M100, 34th edition (Clinical and Laboratory Standards Institute, 2024). No automated antimicrobial susceptibility testing system was used in this study.

### Pyocin activity by overlay spot plate assay

The antibacterial activity of pyocins against MDR/XDR *P. aeruginosa* strains was assessed using an overlay spot plate assay. Eighty microliters of mid-log-phase bacterial culture (OD_600_ = 0.6) was mixed with eight mL of 0.8% (w/v) molten soft agar and overlaid onto Luria–Bertani (LB) agar plates. Five microliters of purified pyocin (200 µg/mL) was spotted onto the solidified overlays. Plates were incubated at 37 °C for 24 h, and the presence of clear inhibition zones was recorded as evidence of bactericidal activity.

### Pyocin minimum inhibitory concentration determination

The MIC of each pyocin was determined using twofold dilution series ranging from 200 µg/mL to 0.2 µg/mL. Five microliters of each dilution was spotted onto overlay plates prepared as described above and incubated at 37 °C for 24 h. The MIC was defined as the lowest pyocin concentration that produced a visible zone of inhibition.

### Biofilm formation screening

Biofilm formation was assessed using a crystal violet staining assay. Overnight cultures of *P. aeruginosa* were inoculated into sterile 96-well polystyrene microtiter plates containing 100 µL of tryptic soy broth (TSB) per well and incubated at 37 °C for 20 h under static conditions. Following incubation, planktonic cells were removed and wells were gently washed three times with deionized water. Plates were air-dried for 1 h at room temperature and stained with 125 µL of 0.1% (w/v) crystal violet solution for 10 min. Excess stain was removed and bound dye was solubilized with 125 µL of 30% (v/v) acetic acid. Absorbance was measured at 550 nm using a microplate reader. All assays were performed in quadruplicate (O’Toole, 2011).

### Biofilm eradication assay

Established biofilms were treated with pyocins to assess their biofilm eradication activity. Biofilms were grown as described above by incubating bacterial cultures at 37 °C for 20 h. Following incubation, planktonic cultures were carefully removed, and wells were washed three times with sterile normal saline to eliminate non-adherent cells. Residual liquid was gently removed by blotting the inverted plate on sterile absorbent paper. Subsequently, 125 µL of pyocin solutions at varying concentrations were added to each well and incubated at 37 °C for 1 h. After treatment, pyocin solutions were removed and wells were washed three times with sterile normal saline. Biofilm metabolic activity was then assessed using the reduction of 2,3,5-triphenyl tetrazolium chloride (TTC). Briefly, 125 µL of 0.1% TTC prepared in TSB supplemented with 0.2% glucose was added to each well, followed by incubation at 37 °C in the dark with shaking at 120 rpm for 5 h. Following incubation, 100 µL of the reduced dye was transferred to a new flat-bottom microtiter plate, and absorbance was measured at 405 nm. Assays were conducted in quadruplicate (Sabaeifard et al., 2014).

### Genome dataset collection and identification of bacteriocin-related genes

Whole-genome sequences of clinical *P. aeruginosa* isolates from Thailand were retrieved from the National Center for Biotechnology Information (NCBI) Pathogen Detection database and supplemented with in-house genome data generated through a previous study (Wankaew et al., 2026), corresponding to the isolates used for pyocin susceptibility testing in this study. Multilocus sequence typing (MLST) profiles were assigned according to the PubMLST scheme (Maiden et al., 1998). Phylogenetic analysis was performed using *P. aeruginosa* PAO1 (NC_002516.2) as the reference genome. Core single nucleotide polymorphisms (SNPs), including substitutions, deletions, and insertions, were identified using Snippy v4.6.0 (https://github.com/tseemann/snippy). Maximum likelihood phylogenetic inference was conducted using IQ-TREE v. 2.4.0 with the GTR + G model and 1,000 bootstrap replicates (Minh et al., 2020). The final tree was visualized and annotated using iTOL v7 (Letunic & Bork, 2024).

Reference sequences for known genes encoding pyocins, outer membrane transporters, and associated immunity proteins were curated from published literature. A summary of the curated pyocins, including their cytotoxic activity, outer membrane transporters, and supporting references, is provided in Table S1. Accession numbers for the pyocin-related reference genes are listed in Table S2. Homologous genes were identified within the genome dataset using BLASTp and tblastn searches, applying thresholds of ≥90% sequence identity and ≥90% query coverage to ensure reliable detection of homology. The prevalence and distribution of pyocin-related genes were analyzed in relation to sequence types (STs). Associations between pyocin-related gene presence and *P. aeruginosa* STs were evaluated using chi-square tests with Benjamini–Hochberg false discovery rate (FDR) correction. Analyses were restricted to ST groups comprising five or more isolates to ensure statistical robustness. Gene presence/absence was binarized (1/0) prior to analysis. All statistical analyses were performed in R v.4.3.1 (R Core Team, 2025).

### Ethics statement

This study used bacterial isolates obtained from an existing culture collection and publicly available bacterial genome sequences. No human participants were recruited, and no identifiable patient information was accessed or analyzed in this study.

## Results

### Susceptibility of MDR/XDR *P. aeruginosa* isolates to pyocins

The *P. aeruginosa* isolates analyzed in this study constitute a genetically diverse collection of MDR/XDR clinical strains, as previously characterized (Wankaew et al., 2026). MLST revealed a broad distribution of STs, including several internationally recognized high-risk clones, such as ST235, ST357, and ST654.

Phenotypic antimicrobial susceptibility testing confirmed that all isolates met the criteria for MDR, with a substantial proportion additionally classified as XDR. Notably, isolates PATH-2 (ST3390), PATH-23 (ST233), PATH-25 (ST357), and PATH-42 (ST654), together with multiple isolates belonging to ST5340 (PATH-4, PATH-31, PATH-34, PATH-46, PATH-47, and PATH-48), exhibited pan-resistance to all 10 tested antipseudomonal agents, including colistin (Fig. 1). These findings highlight the extremely limited therapeutic options currently available for a subset of circulating clinical *P. aeruginosa* strains.

**Figure 1:**
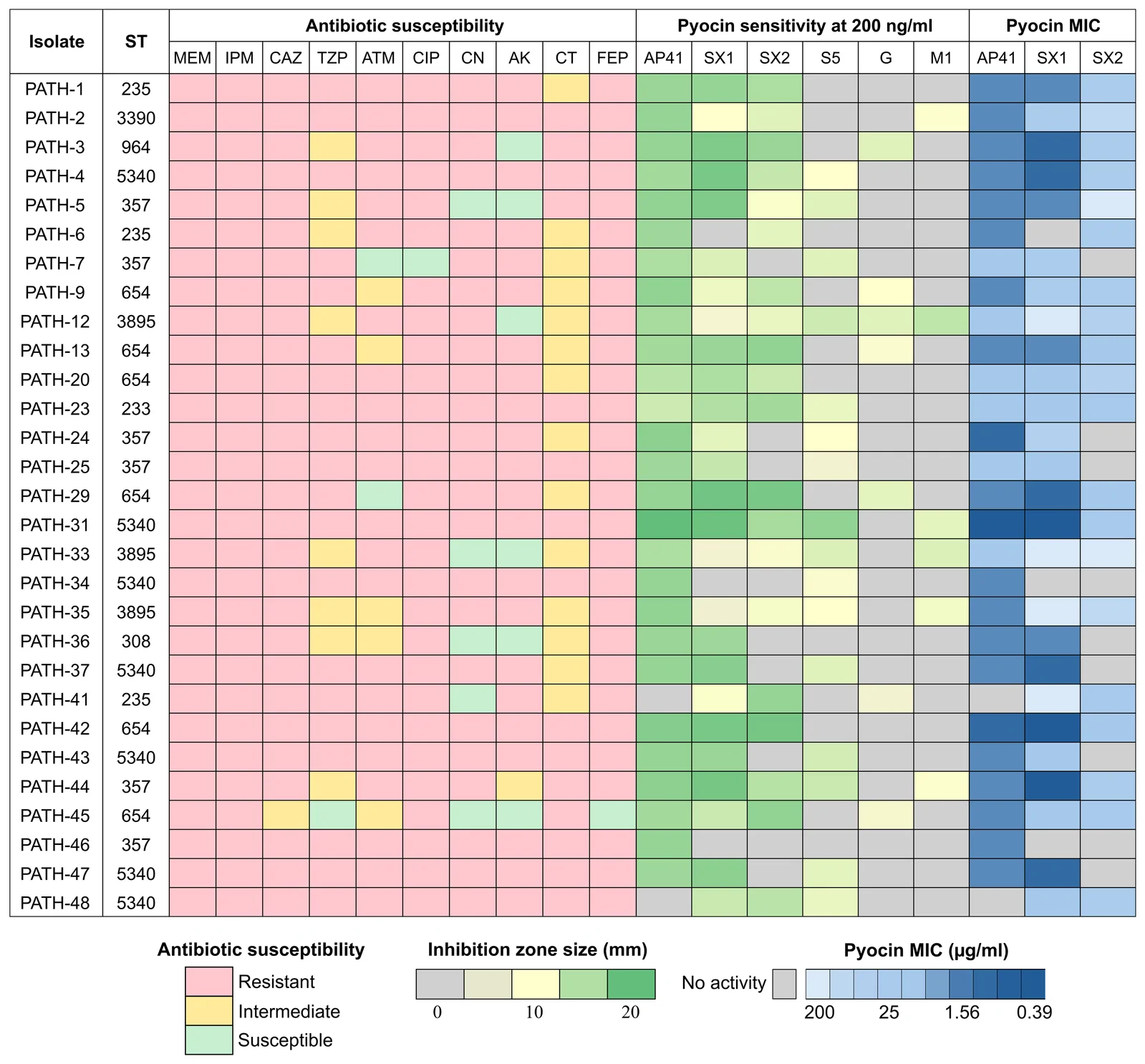
Sequence types, antibiotic resistance patterns, and susceptibility to pyocins among MDR and XDR *P. aeruginosa* clinical isolates from Thailand. Antimicrobial susceptibility profiles were determined by disk diffusion according to CLSI guidelines, with colistin susceptibility assessed by broth microdilution. Antibiotic abbreviations: CIP, ciprofloxacin; CT, colistin; CN, gentamicin; AK, amikacin; ATM, aztreonam; MEM, meropenem; IPM, imipenem; FEP, cefepime; TZP, piperacillin–tazobactam; CAZ, ceftazidime. Pyocin susceptibility was evaluated at 200 µg/mL using an overlay spot assay. Inhibition zones surrounding the pyocin spots were measured in millimeters after 24 h of incubation. MICs of pyocins were determined using twofold serial dilutions (200 to 0.2 µg/mL) in the same overlay system. The MIC was defined as the lowest concentration at which a visible inhibition zone was observed after incubation.

To assess the activity of pyocins against this highly resistant panel, initial screening was performed using an overlay spot assay at a fixed concentration of 200 µg/mL. Of the six S-type pyocins evaluated (AP41, SX1, SX2, S5, G and M1), AP41, SX1, and SX2 demonstrated the broadest and most potent bactericidal activity. AP41 showed the widest inhibitory spectrum, with activity against 93.1% of the isolates, followed by SX1 (89.7%) and SX2 (69.0%). In contrast, pyocins S5, G and M1 exhibited more limited and strain-specific activity across the isolate collection (Fig. 1).

Based on these findings, AP41, SX1, and SX2 were selected for further quantitative evaluation. MIC assays confirmed the high potency of these pyocins and were concordant with the qualitative screening results. In particular, AP41 and SX1 displayed exceptionally strong antibacterial activity, with MIC values reaching the nanogram-per-milliliter range for multiple isolates (Fig. 1). Notably, this potent activity was observed even against XDR strains resistant to last-resort antibiotics, including colistin. Collectively, these results highlight the substantial antibacterial efficacy of selected pyocins against genetically and phenotypically diverse MDR/XDR *P. aeruginosa* isolates, supporting their potential as alternative options for the treatment of recalcitrant infections.

### Pyocin-mediated eradication of established biofilms in high-biofilm-forming MDR/XDR *P. aeruginosa*

Screening of *P. aeruginosa* isolates using a microtiter plate crystal violet staining assay revealed substantial variability in biofilm-forming capacity. Three isolates, namely PATH-3, PATH-42, and PATH-45, consistently exhibited the highest levels of biofilm formation and were therefore selected for subsequent biofilm eradication assays (Fig. 2).

**Figure 2:**
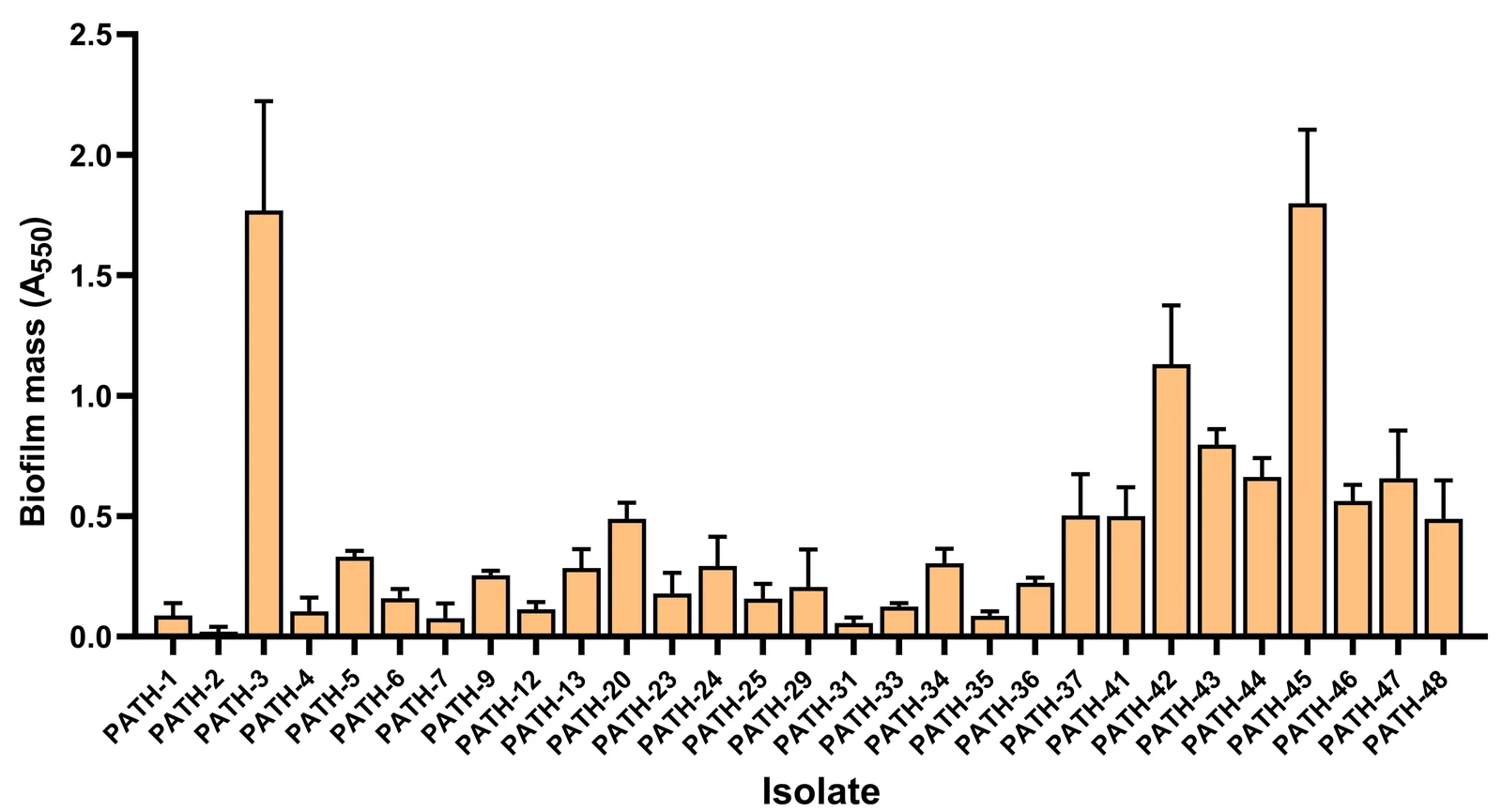
Screening of biofilm formation among MDR/XDR *P. aeruginosa* isolates. Biofilm formation was assessed using a crystal violet microtiter plate assay in 96-well plates with tryptic soy broth, incubated at 37 °C for 20 h under static conditions. Biofilm biomass was quantified by measuring absorbance at 550 nm. Data represent the mean ± standard deviation of four replicates.

Pyocin-mediated eradication of established biofilms was evaluated using three high-biofilm-forming MDR/XDR *P. aeruginosa* isolates exposed to increasing concentrations of three pyocins (AP41, SX1, and SX2) and two aminoglycoside antibiotics (gentamicin and amikacin) (Fig. 3). All three pyocins demonstrated concentration-dependent biofilm eradication across the tested isolates, with marked reductions in the metabolic activity of biofilm-associated cells observed at higher concentrations.

**Figure 3:**
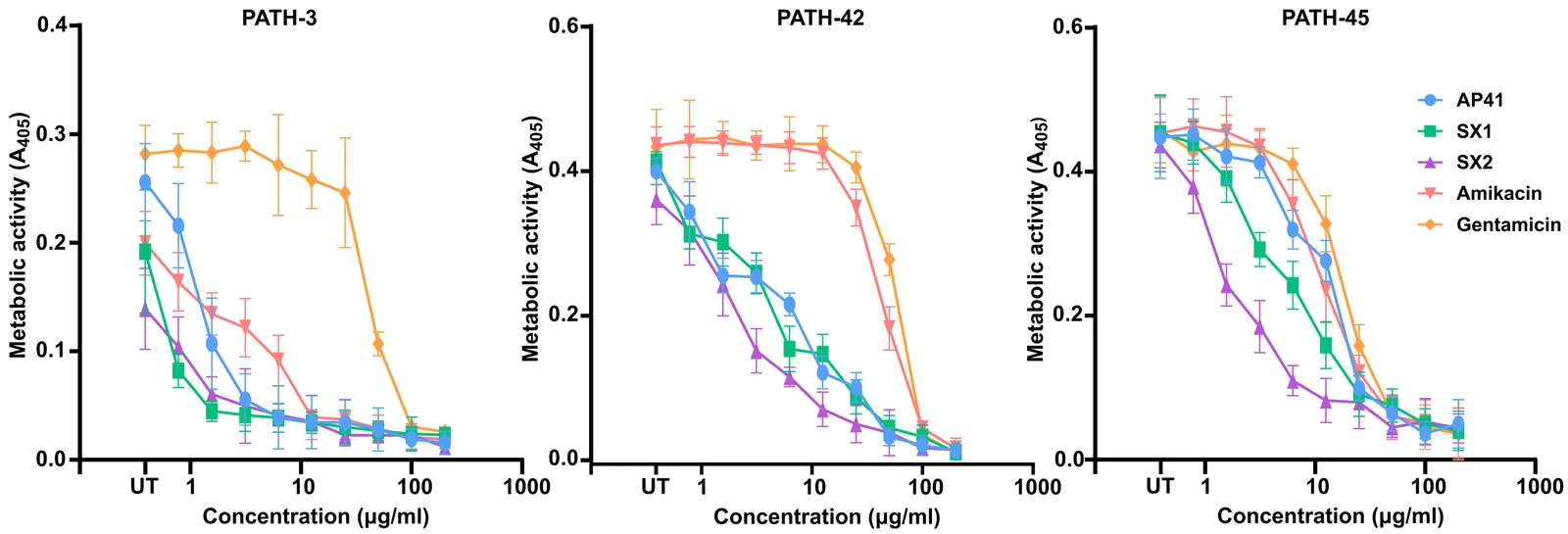
Dose-dependent eradication of established biofilms by pyocins and comparator antibiotics in high-biofilm-forming MDR/XDR *P. aeruginosa* isolates. Biofilms were formed in 96-well plates using tryptic soy broth at 37 °C for 20 h under static conditions and subsequently treated with pyocins or antibiotics at varying concentrations for 1 h. Biofilm metabolic activity was quantified using a TTC reduction assay by measuring absorbance at 405 nm. Nonzero pyocin and antibiotic concentrations are presented on a log_10_ scale. UT represents the untreated control (zero µg/mL) and is displayed separately from the logarithmically scaled concentrations. Data represent the mean ± standard deviation of four replicates.

In PATH-3, which was resistant to gentamicin but susceptible to amikacin, pyocin treatment resulted in substantial biofilm eradication, whereas gentamicin showed limited activity against established biofilms. In PATH-42, which was resistant to both gentamicin and amikacin, pyocins retained strong antibiofilm activity, while both antibiotics exhibited minimal biofilm eradication across the concentration range tested. In PATH-45, which was susceptible to both gentamicin and amikacin in antimicrobial susceptibility testing, both antibiotics and pyocins produced dose-dependent reductions in biofilm metabolic activity, with pyocins SX1 and SX2 showing reductions in biofilm metabolic activity at concentrations lower than those required for gentamicin and amikacin. Collectively, these results demonstrate that pyocins AP41, SX1, and SX2 exhibit robust antibiofilm activity against established biofilms formed by MDR/XDR *P. aeruginosa*.

### Phylogenetic distribution of pyocin-associated genes among Thai *P. aeruginosa* genomes

An SNP-based phylogenetic tree was created to provide an overview of the evolution of the dataset, with branch support indicated by bootstrap values. A gene present/absent heatmap was also produced to illustrate the distribution of pyocin transporter genes across the phylogeny (Fig. 4). The analysis included 105 Thai *P. aeruginosa* genomes retrieved from the NCBI Pathogen Detection database, supplemented with genomes generated in our previous study (Wankaew et al., 2026). Annotation of STs highlighted multiple dominant clonal lineages, including ST235, ST357, and ST654, which were consistent with the observed phylogenetic clustering and enabled comparison between phylogenetic structure and pyocin-associated gene profiles. A clear distinction was observed between broadly conserved and lineage-restricted pyocin uptake systems. Two transporter genes, *fptA* and *crtA*, were detected in all isolates. The genes *fiuA* and *hur* were present in nearly all isolates (>95%), indicating that the genomic capacity for pyocin uptake *via* these transporters is widely conserved across diverse lineages. In contrast, the ferripyoverdine receptor genes *fpvAI* and *fpvAII* were not universally conserved among isolates. This suggests that pyocins utilizing these transporters for cellular uptake may exhibit a restricted strain-killing spectrum.

**Figure 4:**
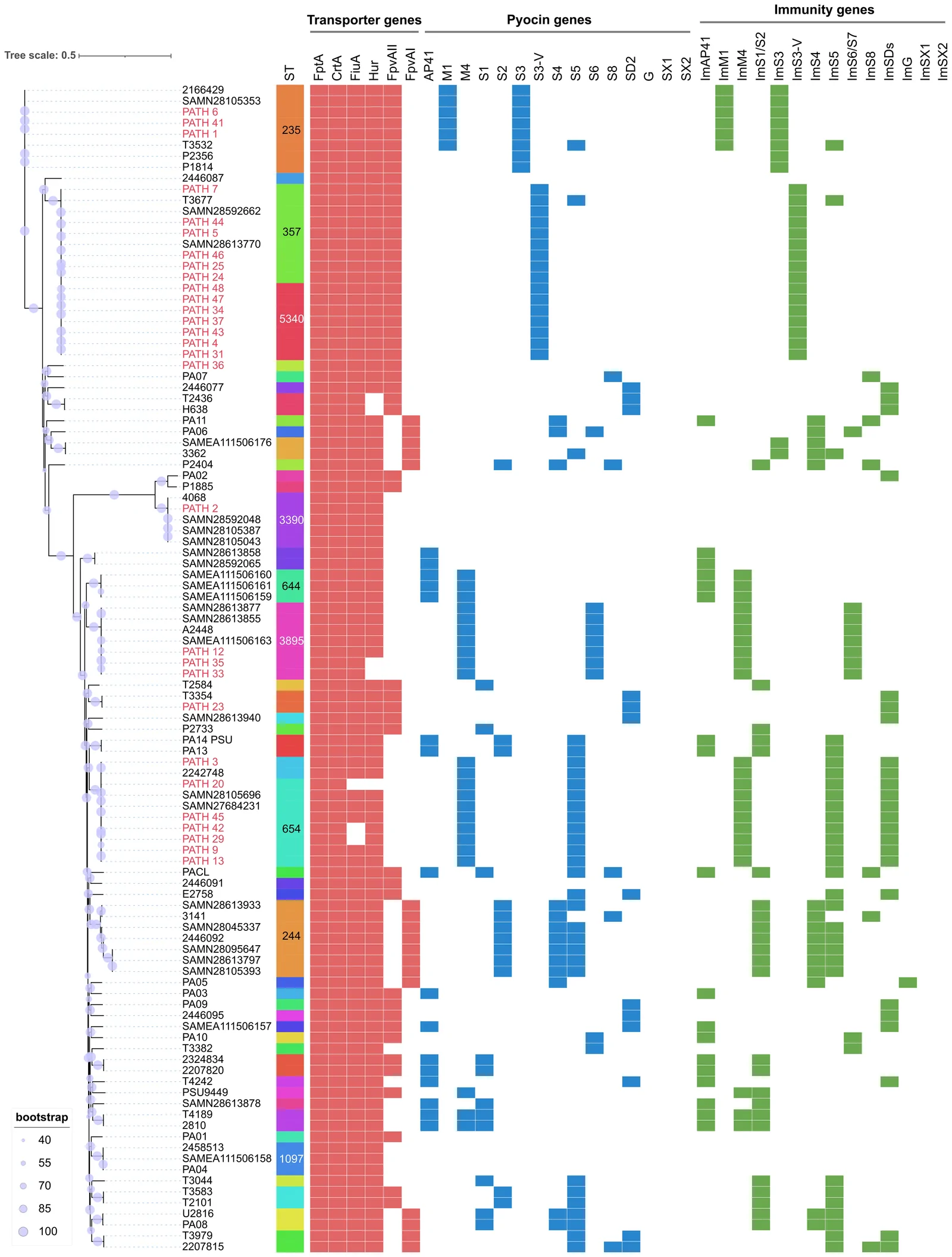
SNP-based phylogeny of Thai *P. aeruginosa.* genomes available in the NCBI Pathogen Detection database annotated with pyocin-associated genes. Isolates used in this study are highlighted in red. Bootstrap support values are shown at key nodes. ST, sequence type.

For S-type pyocin structural genes and their corresponding immunity determinants, gene-per-genome analysis revealed substantial heterogeneity. Among S-type pyocin genes, 35% of genomes encoded one pyocin gene, 35% encoded two, 15% encoded three, and 1% encoded four, whereas 14% lacked detectable S-type pyocin genes. A comparable distribution was observed for immunity genes, with 31% of genomes carrying one determinant, 29% carrying two, 27% carrying three, and 1% carrying four, while 12% carried none. These findings indicate that most *P. aeruginosa* genomes harbor multiple S-type pyocin-related elements, consistent with the species’ diverse bacteriocin repertoires. This study also identified a pyocin S3 variant that shares 95% amino acid identity with canonical pyocin S3 but contains distinct substitutions within the cytotoxic domain. This variant is accompanied by a putative novel immunity protein located immediately downstream, which is genetically distinct from the known S3 immunity protein (39.6% amino acid identity) (Fig. 5). Both the variant pyocin and its associated immunity gene were strictly lineage-specific, occurring only in ST357 and ST5340. This variant was therefore designated pyocin S3-V for further analysis.

**Figure 5:**
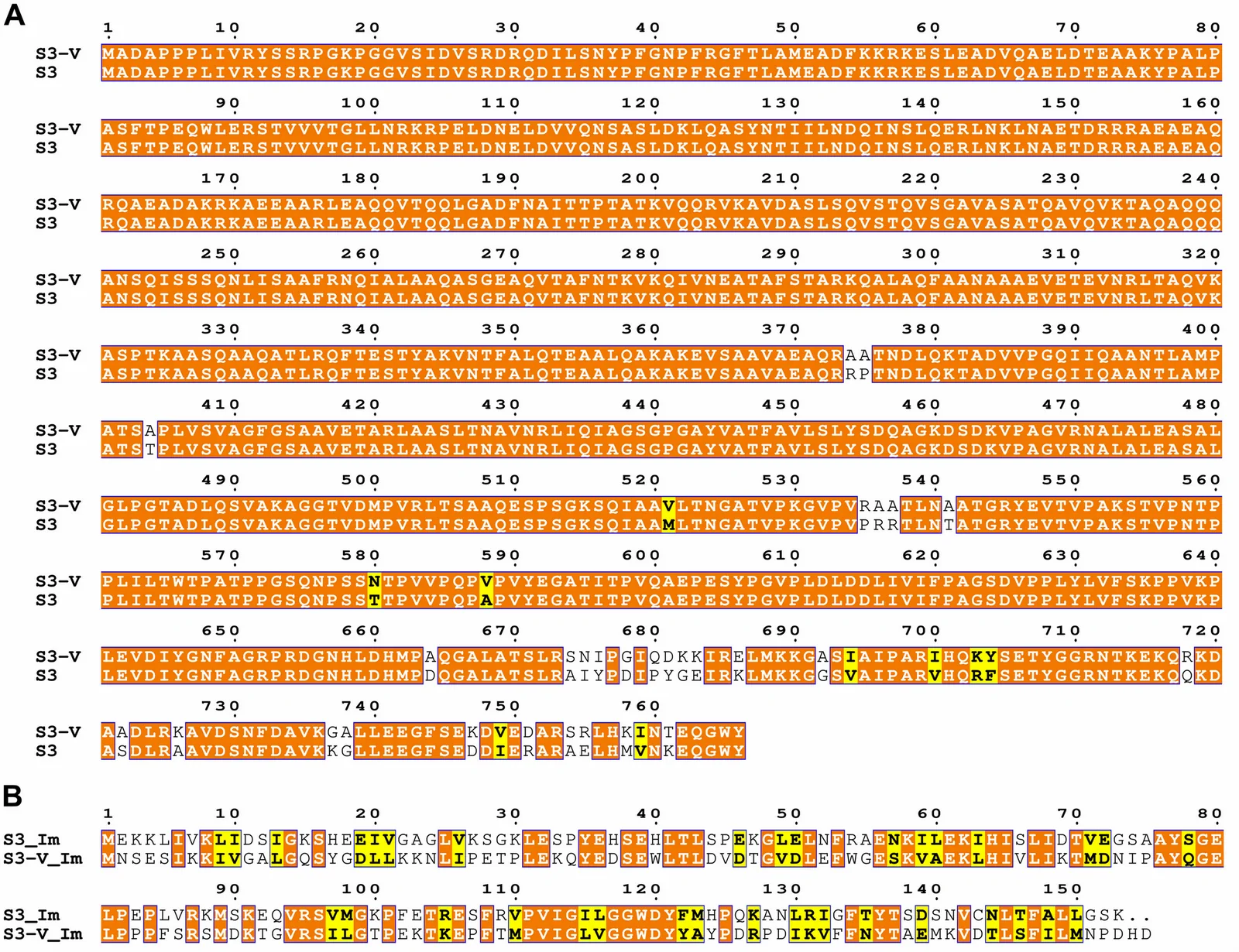
Amino acid sequence alignment of pyocin S3 and the novel pyocin S3 variant (S3-V) and their corresponding immunity proteins. (A) Alignment of the pyocin S3-V and pyocin S3 proteins. (B) Alignment of the S3-V immunity protein (S3-V_Im) and the pyocin S3 immunity protein (S3_Im).

The most abundant pyocins in Thai clinical *P. aeruginosa* isolates were S5 (28.3%), M4 (21.7%), AP41 (15.1%), and S3-V (15.1%). Lower frequencies were observed for S4 (12.3%), SDs (12.3%), S2 (11.3%), S1 (10.4%), S6 (9.4%), M1 (5.7%), and S8 (4.7%). No genomes carried pyocins G, SX1, or SX2 (Fig. 4). The prevalence of genes encoding immunity determinants largely mirrored that of the genes encoding their corresponding pyocins. Genes conferring cognate immunity to S5 (28.3%), S1/S2 (22.6%), SDs (22.6%), and M4 (21.7%) were among the most common. Moderate frequencies were observed for the genes conferring cognate immunity to S3-V (14.2%), S4 (15.1%), S3 (9.4%), S6/S7 (9.4%), and S8 (5.7%). None carried genes conferring immunity to G, SX1, or SX2 (Fig. 4).

Significant lineage-associated patterns were identified for multiple structural and immunity genes, as demonstrated by chi-square tests assessing associations between gene presence/absence and STs (Table S3). These associations are visually supported by the phylogeny, in which distinct clades exhibit conserved and characteristic pyocin and immunity gene profiles. Collectively, the data demonstrate that, while pyocin uptake machinery is broadly conserved, structural and immunity gene repertoires are highly variable and strongly structured by evolutionary lineage (Fig. 4).

## Discussion

The present study demonstrates that selected pyocins exhibit potent antibacterial activity against MDR and XDR *P. aeruginosa* at both planktonic and biofilm stages, underscoring their potential as alternative or adjunctive antimicrobial agents. Importantly, pyocin activity was observed across isolates representing diverse genetic backgrounds, indicating that susceptibility is not restricted to specific lineages. Among the tested pyocins, AP41, SX1, and SX2 displayed consistent activity against both planktonic and biofilm-associated cells. These findings are consistent with previous reports describing the antibiofilm activity of pyocin S2 against *P. aeruginosa*, including established biofilms that exhibit tolerance to antibiotic treatment (Smith et al., 2012). The efficacy against biofilm-associated cells is particularly notable, as biofilm-associated *P. aeruginosa* is notoriously tolerant or resistant to conventional antibiotics due to reduced metabolic activity, restricted drug penetration, and enhanced stress response within biofilms (Thi, Wibowo & Rehm, 2020; Tuon et al., 2022). In contrast to small-molecule antibiotics, pyocins exert their activity through highly specific receptor-mediated uptake followed by intracellular killing mechanisms, such as nuclease or pore-forming activity, which are largely independent of classical antibiotic targets (Michel-Briand & Baysse, 2002). As a result, resistance mechanisms that compromise antibiotic efficacy, such as *β*-lactamase production, target modification, or efflux pump overexpression, are unlikely to impair pyocin activity (Smith et al., 2012). Collectively, these findings suggest that pyocins can effectively target *P. aeruginosa* cells embedded within biofilms, highlighting their potential value in the treatment of chronic and device-associated infections.

Analysis of pyocin gene distribution provided critical genomic context for interpreting the susceptibility patterns observed in this study. The Thai clinical isolates investigated here lacked both the gene encoding the AP41 pyocin and the gene conferring immunity to it, which is consistent with the broad antibacterial activity of AP41 observed in susceptibility testing. However, extension of the analysis to a broader genomic dataset revealed that a subset of isolates (15.1%) harbored both such genes. The presence of cognate immunity determinants suggests that AP41-mediated killing may be ineffective against specific lineages due to immunity-based resistance. This underscores the importance of incorporating population-level genomic data when evaluating bacteriocins for therapeutic applications, as the possession of immunity genes can directly influence pyocin efficacy (Ghequire & De Mot, 2014). Notably, SX1 and SX2 exhibited a favorable genomic profile characterized by the widespread presence of a conserved transporter gene (*crtA*) (Premsuriya et al., 2023), coupled with the absence of genes encoding these pyocins or determinants of immunity to them in the Thai clinical *P. aeruginosa* population. The rarity of endogenous SX1 and SX2 immunity determinants suggests that pre-existing resistance mechanisms targeting these pyocins are likely to be minimal, supporting their potential suitability as therapeutic candidates.

However, despite the presence of predicted pyocin transporter genes and the absence of genes encoding pyocins or cognate immunity determinants, a subset of isolates remained resistant to pyocins, including G, SX1, and SX2. This discordance between genomic predictions and actual susceptibility suggests that resistance mechanisms beyond the classical absence of cognate transporters or presence of immunity-mediated protection may contribute to reduced pyocin susceptibility. One plausible explanation involves extracellular degradation of pyocins by secreted proteases. S-type pyocins are recognized for their sensitivity to proteolytic degradation (Ghequire & De Mot, 2014). *P. aeruginosa* produces several extracellular proteases including LasB elastase, which is particularly abundant and is known for its broad substrate specificity and capacity to degrade host structural proteins, immune factors, and antimicrobial peptides (Everett & Davies, 2021). Protease-mediated inactivation, particularly by LasB elastase, may limit the stability and activity of exogenously applied pyocins. In addition, LasB is a well-recognized virulence factor that contributes to tissue damage, biofilm development, and immune evasion, which can adversely impact treatment outcomes (Everett & Davies, 2021). Consequently, previous studies have explored the development of LasB inhibitors as potential antivirulence agents and have shown that pharmacological inhibition of LasB can reduce bacterial virulence (Cathcart et al., 2011; Everett & Davies, 2021; Konstantinović et al., 2023). Given the findings of this study, co-administration of pyocins with protease inhibitors, particularly LasB inhibitors, may represent a rational strategy to enhance pyocin stability and potentially improve antimicrobial efficacy (Cathcart et al., 2011; Everett & Davies, 2021).

Despite the promising findings, several limitations of this study warrant consideration. First, the phenotypic evaluation was conducted using a relatively small, selected panel of MDR/XDR *P. aeruginosa* isolates, which may not fully represent the diversity of clinical *P. aeruginosa* circulating in Thailand. In addition, antibiofilm activity was evaluated using only three strong biofilm-producing isolates in an *in vitro* model. The observed discordance between genomic predictors and actual pyocin susceptibility also suggests the presence of unresolved resistance mechanisms. Future studies incorporating larger and more diverse collections of clinical isolates, mechanistic investigations of pyocin resistance, *in vivo* validation, and rational pyocin engineering will be essential for developing candidates with enhanced stability, broader activity spectra, and improved therapeutic potential.

## Conclusion

This study demonstrates that pyocins exhibit potent antibacterial activity against MDR and XDR *P. aeruginosa*, with efficacy observed in both planktonic and biofilm-associated states. The efficacy of AP41, SX1, and SX2 against genetically diverse MDR/XDR isolates highlights their potential for development as alternative or adjunctive antimicrobial agents. Nevertheless, variability in pyocin susceptibility among isolates, including resistance observed despite the presence of predicted transporter genes and absence of cognate immunity determinants, highlights the need for further mechanistic investigation.

##  Supplemental Information

10.7717/peerj.21850/supp-1 Data S1 Raw data for pyocin assays

10.7717/peerj.21850/supp-2 Table S1 Summary of curated pyocins, including cytotoxic activity, outer membrane transporter, and supporting references

10.7717/peerj.21850/supp-3 Table S2 Accession numbers of reference sequences for pyocin-related genes used in this study

10.7717/peerj.21850/supp-4 Table S3 Chi-square test results evaluating the association between gene presence/absence of pyocin-related genes and sequence types (STs), with Benjamini–Hochberg FDR correction Only statistically significant associations (FDR-adjusted *p* < 0.05) are shown.

## Additional Information and Declarations

### Data Availability

The following information was supplied regarding data availability: The raw measurements are available in the Supplemental Files.
