# Supplementary material for: Evaluation of pyocin activity and pyocin-related gene distribution in multidrug- and extensively drug-resistant Pseudomonas aeruginosa isolates from Thailand
Source: PeerJ. 2026 Oct 21;14:e21850. doi: 10.7717/peerj.21850 (PMC13695419; doi:10.7717/peerj.21850)
Supplement: Supplementary file 1 [file peerj-14-21850-s002.docx]

**Table S1.** Summary of curated pyocins, including cytotoxic activity, outer membrane transporter, and supporting references.

| Pyocin | Cytotoxic activity | Transporter | Reference |
| --- | --- | --- | --- |
| S1 | DNase (HNH) | Hur | (Ito et al, 1970) |
| S2 | DNase (HNH) | FpvAI | (Ohkawam et al, 1973)  (Denayer et al, 2007) |
| S3 | DNase | FpvAII | (Duport et al,1995)  (de Chial et al., 2003) |
| S4 | tRNAse | FpvAI | (Parret & De Mot, 2000)  (Elfarash et al, 2012) |
| S5 | Pore-forming | FptA | (Parret & De Mot, 2000)  (Elfarash et al., 2014) |
| S6 | rRNase | Hur | (Dingemans et al., 2016) |
| S8 | DNase (HNH) | Unknown | (Turano et al., 2017) |
| AP41 | DNase (HNH) | Unknown | (Sano & Kageyama, 1981) |
| PaeM1 | Lipid II degradation | FiuA | (Barreteau et al., 2012) (Latino et al., 2019) |
| PaeM2 | Lipid II degradation | FiuA | (Latino et al., 2019) |
| PaeM4 | Lipid II degradation | Hur | (Ghequire & Öztürk, 2018) |
| SD1 | tRNase | Hur | (McCaughey et al., 2016) |
| SD2 | tRNase | FpvAI | (McCaughey et al., 2016) |
| SD3 | DNase | FpvAII | (McCaughey et al., 2016) |
| G | Unknown | Hur | (Atanaskovic et al., 2020) |
| SX1 | DNase | CrtA | (Premsuriya et al., 2023) |
| SX2 | Unknown | CrtA | (Premsuriya et al., 2023) |

**Reference**

Atanaskovic I, Mosbahi K, Sharp C, Housden NG, Kaminska R, Walker D, Kleanthous C. 2020. Targeted killing of *Pseudomonas aeruginosa* by pyocin G occurs via the hemin transporter Hur. *Journal of Molecular Biology* 432:3869–3880. DOI: 10.1016/j.jmb.2020.04.020.

Barreteau H, Tiouajni M, Graille M, Josseaume N, Bouhss A, Patin D, Blanot D, Fourgeaud M, Mainardi J-L, Arthur M, van Tilbeurgh H, Mengin-Lecreulx D, Touzé T. 2012. Functional and structural characterization of PaeM, a colicin m-like bacteriocin produced by *Pseudomonas aeruginosa*. *Journal of Biological Chemistry* 287:37395–37405. DOI: 10.1074/jbc.M112.406439.

de Chial M, Ghysels B, Beatson SA, Geoffroy V, Meyer JM, Pattery T, Baysse C, Chablain P, Parsons YN, Winstanley C, Cordwell SJ, Cornelis P. 2003. Identification of type II and type III pyoverdine receptors from *Pseudomonas aeruginosa*. *Microbiology* 149:821–831. DOI: 10.1099/mic.0.26136-0.

Denayer S, Matthijs S, Cornelis P. 2007. Pyocin S2 (Sa) kills *Pseudomonas aeruginosa* strains via the FpvA type I ferripyoverdine receptor. *Journal of Bacteriology* 189:7663–7668. DOI: 10.1128/JB.00992-07.

Dingemans J, Ghequire MGK, Craggs M, De Mot R, Cornelis P. 2016. Identification and functional analysis of a bacteriocin, pyocin S6, with ribonuclease activity from a *Pseudomonas aeruginosa* cystic fibrosis clinical isolate. *MicrobiologyOpen* 5:413–423. DOI: 10.1002/mbo3.339.

Duport C, Baysse C, Michel-Briand Y. 1995. Molecular characterization of pyocin S3, a novel s-type pyocin from *Pseudomonas aeruginosa*. *Journal of Biological Chemistry* 270:8920–8927. DOI: 10.1074/jbc.270.15.8920.

Elfarash A, Dingemans J, Ye L, Hassan AA, Craggs M, Reimmann C, Thomas MS, Cornelis P. 2014. Pore-forming pyocin S5 utilizes the FptA ferripyochelin receptor to kill *Pseudomonas aeruginosa*. *Microbiology* 160:261–269. DOI: 10.1099/mic.0.070672-0.

Elfarash A, Wei Q, Cornelis P. 2012. The soluble pyocins S2 and S4 from *Pseudomonas aeruginosa* bind to the same FpvAI receptor. *MicrobiologyOpen* 1:268–275. DOI: 10.1002/mbo3.27.

Ghequire MGK, Öztürk B. 2018. A Colicin M-type bacteriocin from *Pseudomonas aeruginosa* targeting the HxuC heme receptor requires a novel immunity partner. *Applied and Environmental Microbiology* 84. DOI: 10.1128/AEM.00716-18.

Ito S, Kageyama M, Egami F. 1970. Isolation and characterization of pyocins from several strains of *Pseudomonas aeruginosa*. *The Journal of General and Applied Microbiology* 16:205–214. DOI: 10.2323/jgam.16.3_205.

Latino L, Patin D, Chérier D, Touzé T, Pourcel C, Barreteau H, Mengin-Lecreulx D. 2019. Impact of FiuA outer membrane receptor polymorphism on the resistance of *Pseudomonas aeruginosa* toward peptidoglycan lipid ii-targeting PaeM pyocins. *Journal of Bacteriology* 201. DOI: 10.1128/JB.00164-19.

McCaughey LC, Josts I, Grinter R, White P, Byron O, Tucker NP, Matthews JM, Kleanthous C, Whitchurch CB, Walker D. 2016. Discovery, characterization and *in vivo* activity of pyocin SD2, a protein antibiotic from *Pseudomonas aeruginosa*. *Biochemical Journal* 473:2345–2358. DOI: 10.1042/BCJ20160470.

Ohkawam I, Kageyama M, Egami F. 1973. Purification and properties of pyocin S2. *The Journal of Biochemistry* 73:281–289.

Parret A, De Mot R. 2000. Novel bacteriocins with predicted tRNase and pore‐forming activities in *Pseudomonas aeruginosa* PAO1. *Molecular Microbiology* 35:472–473. DOI: 10.1046/j.1365-2958.2000.01716.x.

Premsuriya J, Mosbahi K, Atanaskovic I, Kleanthous C, Walker D. 2023. Outer membrane translocation of pyocins via the copper regulated TonB-dependent transporter CrtA. *Biochemical Journal* 480:1035–1049. DOI: 10.1042/BCJ20220552.

Sano Y, Kageyama M. 1981. Purification and properties of an S-type pyocin, pyocin AP41. *Journal of Bacteriology* 146:733–739. DOI: 10.1128/jb.146.2.733-739.1981.

Turano H, Gomes F, Barros-Carvalho GA, Lopes R, Cerdeira L, Netto LES, Gales AC, Lincopan N. 2017. Tn *6350*, a novel transposon carrying pyocin S8 genes encoding a bacteriocin with activity against carbapenemase-producing *Pseudomonas aeruginosa*. *Antimicrobial Agents and Chemotherapy* 61. DOI: 10.1128/AAC.00100-17.
