# Supplementary material for: Evaluation of pyocin activity and pyocin-related gene distribution in multidrug- and extensively drug-resistant Pseudomonas aeruginosa isolates from Thailand
Source: PeerJ. 2026 Oct 21;14:e21850. doi: 10.7717/peerj.21850 (PMC13695419; doi:10.7717/peerj.21850)
Supplement: Supplementary file 2 [file peerj-14-21850-s003.docx]

**Table S2.** Accession numbers of reference sequences for pyocin-related genes used in this study.

| Protein | Function | Accession Number | Database |
| --- | --- | --- | --- |
| S1 | Pyocin | WP_128711198.1 | NCBI |
| S2 | Pyocin | NP_249841.1 | NCBI |
| S3 | Pyocin | CAA54958.1 | NCBI |
| S4 | Pyocin | WP_003148085.1 | NCBI |
| S5 | Pyocin | WP_003115311.1 | NCBI |
| S6 | Pyocin | KYO98147.1 | NCBI |
| S8 | Pyocin | APW77308.1 | NCBI |
| AP41 | Pyocin | BAA02196.1 | NCBI |
| PaeM1 | Pyocin | ERZ09841.1 | NCBI |
| PaeM2 | Pyocin | SKA49653.1 | NCBI |
| PaeM4 | Pyocin | ERY59288.1 | NCBI |
| SD1 | Pyocin | AHC66815.1 | NCBI |
| SD2 | Pyocin | WP_05804.1 | NCBI |
| SD3 | Pyocin | WP_076939003.1 | NCBI |
| G | Pyocin | WP_170835785.1 | NCBI |
| SX1 | Pyocin | UYS84543.1 | NCBI |
| SX2 | Pyocin | UYS84545.1 | NCBI |
| ImS1/S2 | Immunity protein | WP_003120570.1 | NCBI |
| ImS3 | Immunity protein | CAA54959.1 | NCBI |
| ImS4 | Immunity protein | WP_003115172.1 | NCBI |
| ImS5 | Immunity protein | WP_003112562.1 | NCBI |
| ImS6 | Immunity protein | KYO98146.1 | NCBI |
| ImS8 | Immunity protein | APW77309.1 | NCBI |

**Table S2.** (continued).

| Protein | Function | Accession Number | Database |
| --- | --- | --- | --- |
| ImAP41 | Immunity protein | BAA02197.1 | NCBI |
| ImM1 | Immunity protein | WP_126589902.1 | NCBI |
| ImM2 | Immunity protein | WP_128665876.1 | NCBI |
| ImM4 | Immunity protein | WP_170872208.1 | NCBI |
| ImSDs | Immunity protein | WP_016561652.1 | NCBI |
| ImG | Immunity protein | WP_071543135.1 | NCBI |
| ImSX1 | Immunity protein | UYS84544.1 | NCBI |
| ImSX2 | Immunity protein | UYS84546.1 | NCBI |
| FpvAI | Transporter | WP_004349900.1 | NCBI |
| FpvAII | Transporter | AAN62913.1 | NCBI |
| FptA | Transporter | WP_003111969.1 | NCBI |
| FiuA | Transporter | WP_003113264.1 | NCBI |
| Hur | Transporter | WP_003112363.1 | NCBI |
| CrtA | Transporter | WP_003114901.1 | NCBI |
