# Supplementary material for: Evaluation of pyocin activity and pyocin-related gene distribution in multidrug- and extensively drug-resistant Pseudomonas aeruginosa isolates from Thailand
Source: PeerJ. 2026 Oct 21;14:e21850. doi: 10.7717/peerj.21850 (PMC13695419; doi:10.7717/peerj.21850)
Supplement: Supplementary file 3 [file peerj-14-21850-s004.docx]

**Table S3.** Chi-square test results evaluating the association between gene presence/absence of pyocin-related genes and sequence types (STs), with Benjamini–Hochberg FDR correction.

| Gene product | Chi-square | FDR *p*-value |
| --- | --- | --- |
| FpvAI | 51.00 | 4.74E-09 |
| FpvAII | 51.00 | 4.74E-09 |
| M1 | 36.55 | 2.72E-06 |
| M4 | 51.00 | 4.74E-09 |
| S2 | 51.00 | 4.74E-09 |
| S3 | 51.00 | 4.74E-09 |
| S3-A | 51.00 | 4.74E-09 |
| S4 | 51.00 | 4.74E-09 |
| S5 | 38.83 | 1.09E-06 |
| S6 | 51.00 | 4.74E-09 |
| ImM1 | 36.55 | 2.72E-06 |
| ImM4 | 51.00 | 4.74E-09 |
| ImS1/S2 | 51.00 | 4.74E-09 |
| ImS3 | 51.00 | 4.74E-09 |
| ImS3-A | 51.00 | 4.74E-09 |
| ImS4 | 51.00 | 4.74E-09 |
| ImS5 | 38.83 | 1.09E-06 |
| ImS6/S7 | 51.00 | 4.74E-09 |
| ImSDs | 51.00 | 4.74E-09 |

Only statistically significant associations (FDR-adjusted p < 0.05) are shown.
